# Impact of Pericardial Fat on Intracardiac Atrial Impedance and Generator Impedance

**DOI:** 10.1002/joa3.70375

**Published:** 2026-05-27

**Authors:** Shogo Hamaura, Masaomi Kimura, Yuichi Toyama, Kimitaka Nishizaki, Takahiko Kinjo, Yuji Ishida, Taihei Itoh, Shingo Sasaki, Hirofumi Tomita

**Affiliations:** ^1^ Department of Cardiology and Nephrology Hirosaki University Graduate School of Medicine Hirosaki Japan; ^2^ Department of Advanced Management of Cardiac Arrhythmias Hirosaki University Graduate School of Medicine Hirosaki Japan; ^3^ Department of Cardiac Remote Management System Hirosaki University Graduate School of Medicine Hirosaki Japan; ^4^ Department of Advanced Therapeutics for Cardiovascular Diseases Hirosaki University Graduate School of Medicine Hirosaki Japan

**Keywords:** catheter ablation, epicardial adipose tissue, generator impedance, intracardiac atrial impedance

## Abstract

**Background:**

Generator impedance (GI) is a critical parameter that is measured during radiofrequency (RF) ablation. Factors influencing GI and its relationship with intracardiac atrial impedance (IAI) remain unclear; hence, this study aimed to investigate the determinants of IAI and assess their correlation with GI.

**Methods:**

A total of 104 patients who underwent atrial arrythmia ablation were included in this single‐center retrospective study. Pericardial fat (PF) volume was assessed using preoperative contrast‐enhanced computed tomography, and IAI was measured intraoperatively using the BeeAT catheter. The GI was recorded during the first RF application in 48 patients with atrial fibrillation (AF). Multivariate regression analyses were performed to identify the factors influencing IAI and GI.

**Results:**

A positive correlation was observed between the IAI and GI (*n* = 48, *r* = 0.63, *p* < 0.001), and the IAI was an independent predictor of GI by multivariate regression analysis. PF volume (*β* = 0.45, *p* < 0.001), serum sodium levels (*β* = −0.26, *p* = 0.001), and hemoglobin concentrations (*β* = 0.23, *p* = 0.015) were significant determinants of IAI, but not body mass index (BMI). Although PF volume was positively correlated with BMI (*r* = 0.52, *p* < 0.001), the high PF/BMI ratio group had the highest IAI (68.3 ± 10.9 Ω), which significantly exceeded both the low PF/BMI group (53.3 ± 8.5 Ω, *p* < 0.001) and the average PF/BMI group (58.2 ± 8.9 Ω, *p* < 0.001).

**Conclusions:**

IAI was significantly influenced by the PF volume and blood components, and served as a key determinant of GI during AF ablation. These findings suggest that the IAI may be a valuable real‐time indicator for optimizing ablation strategies based on patient‐specific characteristics.

## Graphical abstract

Intracardiac atrial impedance (IAI) was associated with pericardial fat volume, serum sodium levels, and hemoglobin concentrations, but not with body mass index. IAI was also an independent determinant of generator impedance. These findings suggest that IAI may serve as a real‐time indicator for optimizing ablation strategies based on patient‐specific characteristics.

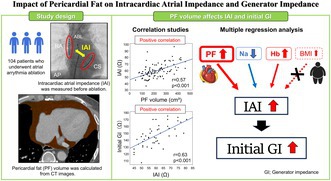

## 1 Introduction

Generator impedance (GI) is a critical parameter in radiofrequency (RF) ablation procedures, representing the total resistance within the RF circuit. This includes various components such as cables, patient patch electrodes, subcutaneous tissue, pericardial tissue, myocardium, and blood in direct contact with the ablation catheter tip [1]. A fundamental understanding of GI is essential because it directly affects the effectiveness of RF energy delivery. When RF energy is applied, GI modulates the amount of current that flows through the catheter, thereby determining the extent of resistive heating and, consequently, the size and depth of the thermal lesion in the myocardium. An increase in GI corresponds to a decrease in the RF current, leading to smaller and less effective lesions. Conversely, a decrease in GI corresponds to an increase in current, producing larger and potentially more effective ablation areas [2]. Therefore, a comprehensive understanding of GI and its contributing factors during RF ablation is critical to ensuring successful outcomes and minimizing potential complications.

Despite its importance, the specific tissue components that influence GI remain underexplored. Pericardial fat (PF) has recently gained attention, primarily due to its association with the development of atrial fibrillation (AF) [3]. PF is the adipose tissue located between the myocardium and the pericardium, and plays a role in various cardiovascular conditions [4]. However, its direct influence on GI during RF ablation has not been fully characterized. Given that PF is non‐conductive, it is plausible that higher volumes of PF could increase the resistance within the RF circuit, thereby affecting GI and the subsequent efficiency of ablation procedures. Furthermore, PF has been correlated with metabolic disorders, such as obesity and insulin resistance, making it an important marker of cardiovascular risk [5, 6, 7].

Another emerging area of interest is the intracardiac atrial impedance (IAI), which is a measurement obtained between the right atrium (RA) and coronary sinus (CS) during atrial cardioversion or ablation. The IAI provides a more localized measure of tissue impedance in the atrial region, and preliminary evidence suggests that it may serve as a surrogate for GI in certain circumstances. Because IAI is measured across the atrial myocardium and adjacent structures, it is sensitive to variations in pericardial tissues, such as PF and blood components. By elucidating the factors that influence IAI, we can gain a deeper understanding of the determinants of GI and refine strategies to optimize ablation efficiency.

The aim of this study was to investigate the key variables influencing IAI, with a particular focus on the role of PF. In addition, we sought to explore the correlation between IAI and GI, with the hypothesis that IAI may serve as a useful predictor of GI during RF ablation procedures.

## 2 Methods

### 2.1 Patients and Data Collection

**FIGURE 1:**
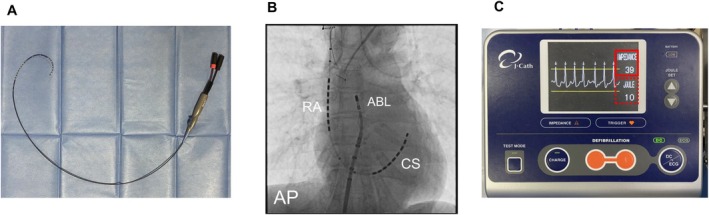
Atrial cardioversion system. (A) Catheter for the atrial cardioversion (BeeAT; Japan Lifeline, Tokyo, Japan). (B) Anterior–posterior view of the configuration of the BeeAT for atrial cardioversion. (C) The intracardiac atrial impedance between the coronary sinus and the right atrium was measured by the BeeAT catheter with a low current of less than 200 μA. If this value is between 21 and 99 Ω (solid red square line), atrial cardioversion system is possible. The output energy can be set between 1 and 30 J (dashed red square line).

The present study was designed as a single‐center, retrospective, observational study conducted between May and November 2020. A total of 121 patients undergoing atrial arrhythmia ablation procedures with an atrial cardioversion system were enrolled. Preoperative contrast‐enhanced cardiac computed tomography (CT) was performed to evaluate PF volume and other anatomical parameters. The IAI was measured during the ablation procedure using a specialized multipolar catheter (BeeAT, Japan Lifeline, Tokyo, Japan) positioned to bridge the RA and CS (Figure 1). This catheter features two distinct electrode arrays, each comprising eight electrodes. A constant current of 700 μA was delivered from the CS electrode array toward the RA electrode array for 1 s to determine the resistance value. To ensure high reproducibility, catheter placement was strictly standardized. The BeeAT catheter was advanced via the internal jugular vein, with the RA electrode array positioned in the superior RA. The CS electrode array was advanced into the coronary sinus such that the proximal‐most electrode of the CS segment was consistently positioned distal to the coronary sinus ostium. This configuration ensured that the entire CS electrode group was contained within the coronary sinus, providing a standardized anatomical circuit for the quantification of intra‐atrial and surrounding tissue impedance.

Patients were excluded if they had incomplete preoperative data, including those without contrast‐enhanced CT scans (*n* = 13). Patients in whom the BeeAT catheter was not properly positioned in the CS during the ablation procedure, which could lead to inaccurate IAI measurements, were also excluded (*n* = 4). Finally, 104 patients were included in the analysis.

Demographic and clinical characteristics were collected for each patient, including age, sex, height, weight, body mass index (BMI), and relevant comorbidities, such as hypertension, diabetes, and history of stroke. Echocardiographic and electrocardiographic data were also collected to assess the left ventricular ejection fraction, left atrial dimension, and atrial volume. Blood chemistry analyses included measurement of the hemoglobin (Hb), electrolytes, brain natriuretic peptide, and creatinine.

Of the 104 patients, 42 had paroxysmal AF, 52 had persistent AF, and 10 had other atrial arrythmias, such as atrial tachycardia and premature atrial contraction. Forty‐eight patients undergoing their first AF ablation with the CARTO3 system (Biosense Webster, Diamond Bar, CA, USA) were selected for further GI analysis. To ensure the reproducibility of GI measurements, a standardized protocol was strictly implemented. The initial GI was recorded at the commencement of the first RF application at the roof of the right superior pulmonary vein (Figure 1B). This site was selected as a stable anatomical landmark representing the highest point in the left atrium. To standardize the contact surface area and minimize variability due to catheter orientation, the catheter was positioned perpendicularly to the tissue with a stable contact force of approximately 10 g in all cases. Furthermore, measurements were consistently taken prior to any previous RF delivery and before significant saline irrigation to eliminate the confounding effects of tissue injury and hemodilution (reduction in hematocrit and increase in electrolytes), both of which can lower the baseline impedance. This subset of patients was used to investigate the relationship between IAI and GI.

### 2.2 Quantification of PF Volume Using Advanced Imaging Techniques

**FIGURE 2:**
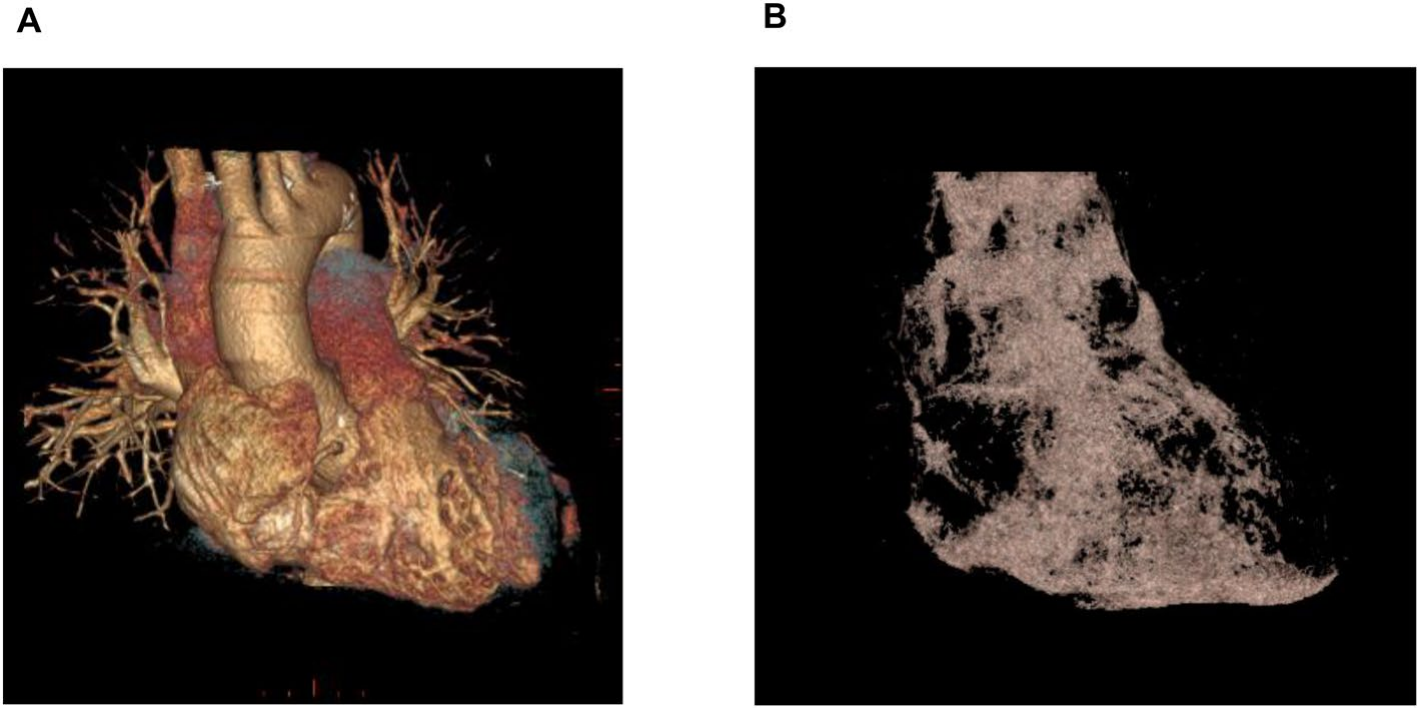
Measurement of pericardial fat (PF) volume. (A) Contrast‐enhanced cardiac computed tomography (CT) with the CT values ranging from −1023 to 3071 HU. (B) Representative constructed CT image with the CT values from −150 to −50 HU indicating PF tissue. HU, Hounsfield unit.

The volume of the PF was accurately quantified using the Advantage Workstation VolumeShare 7 (GE Healthcare, Milwaukee, WI, USA). The contrast‐enhanced CT scans were processed by delineating the region of interest (ROI), which was carefully defined to exclude non‐cardiovascular tissue, and extended anatomically from the aortic arch to the level of the diaphragm. Within this defined ROI, fat tissue was identified based on pixel values within −150 to −50 Hounsfield units (HU), according to established guidelines [8]. To ensure methodological consistency, the PF volume was calculated by applying this specific HU range after manually extracting the cardiovascular structures from the ROI. A representative constructed CT image showing PF tissue is presented in Figure 2.

### 2.3 Ethical Considerations

This study was conducted in accordance with the principles outlined in the Declaration of Helsinki and was approved by the Institutional Review Board of Hirosaki University Graduate School of Medicine (2024‐125). Informed consent was obtained via an opt‐out process.

### 2.4 Statistical Analysis

Continuous variables were expressed as mean ± standard deviation (SD). Group comparisons for continuous variables were performed using one‐way analysis of variance for normally distributed data, followed by Tukey's post hoc test to assess significant differences between groups. A liner regression analysis was performed to examine the correlations between IAI and initial GI or PF volume. Multivariate regression analysis was performed to adjust for variables that were significant in the univariate analysis. Statistical significance was defined as *p* < 0.05. All statistical analyses were conducted using JMP Pro software (version 15.2.0; SAS Institute Inc., Cary, NC, USA).

## 3 Results

### 3.1 Demographic and Clinical Characteristics of the Study Patients

The demographic and clinical characteristics of the study patients are summarized in Table 1. A total of 104 patients were included in the study, of whom 62 (59.6%) were male. The mean age was 65.6 ± 9.9 years, and the mean BMI was 25.1 ± 3.5 kg/m^2^. The mean CHADS_2_ score was 1.3 ± 1.1. In blood chemistry, mean Hb value was 14.4 ± 1.6 g/dL and mean sodium value was 141 ± 2 mEq/L. Echocardiography revealed a mean left ventricular ejection fraction of 60.3% ± 12.9% and a mean left atrial index of 42.1 ± 15.9 mL/m^2^. The mean PF volume was 222.0 ± 97.4 cm^3^ and the mean IAI was 59.2 ± 10.2 Ω.

**TABLE 1:** Baseline characteristics and procedural data of the study patients.

Variables	*n* = 104
Age (years)	65.6 ± 9.9
Male	62 (59.6%)
Height (cm)	163.2 ± 9.6
Weight (kg)	67.5 ± 14.3
BMI (kg/m^2^)	25.1 ± 3.5
CHADS_2_ score	1.3 ± 1.1
Heart failure	15 (14.4%)
Hypertension	69 (66.3%)
Diabetes mellitus	20 (19.2%)
Stroke	8 (7.7%)
Arrhythmia	
PAF	42 (40.4%)
PeAF	52 (50.0%)
Others	10 (9.6%)
Blood chemistry	
Hb (g/dL)	14.4 ± 1.6
BUN (mg/dL)	18 ± 6
Cre (mg/dL)	0.91 ± 0.69
Na (mEq/L)	141 ± 2
HbA1c (%)	6.0 ± 0.7
BNP (pg/mL)	110.7 ± 139.9
Echocardiography	
LVEF (%)	60.3 ± 12.9
LAD (mm)	41.6 ± 6.6
LA volume (mL)	72.7 ± 27.4
LA index (mL/m^2^)	42.1 ± 15.9
PF volume (cm^3^)	222.0 ± 97.4
IAI (Ω)	59.2 ± 10.2

*Note:* Data are given as mean ± standard deviation or number (%).

Abbreviations: BMI, body mass index; BNP, brain natriuretic peptide; BUN, blood urea nitrogen; Cre, creatinine; Hb, hemoglobin; HbA1c, glycated hemoglobin; IAI, intracardiac atrial impedance between the right atrium and the coronary sinus; LA, left atrial; LAD, left atrial dimension; LVEF, left ventricular ejection fraction; Na, sodium; PAF, paroxysmal atrial fibrillation; PeAF, persistent atrial fibrillation; PF, pericardial fat.

### 3.2 Determinants of GI

**FIGURE 3:**
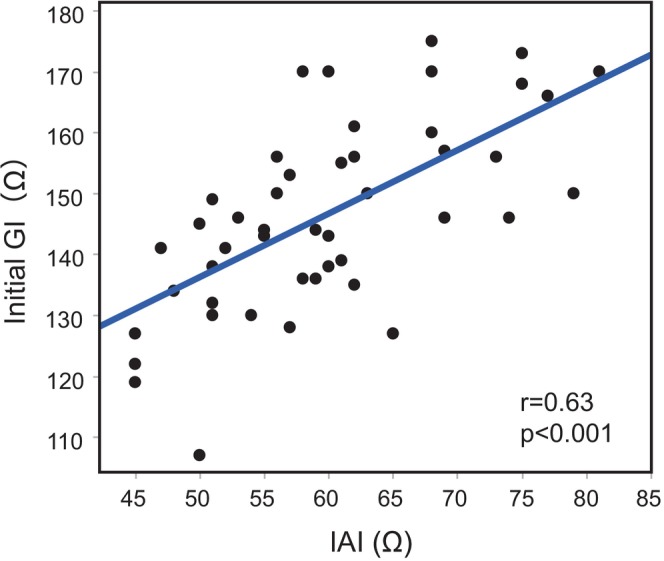
Relationship between the IAI and initial GI. The initial GI was positively correlated with the IAI (*n* = 48, *r* = 0.63, *p* < 0.001). GI, generator impedance; IAI, intracardiac atrial impedance.

The initial GI in the subset of 48 patients who underwent RF ablation using the CARTO3 system was 146.8 ± 15.7 Ω. Figure 3 shows a significant positive correlation between the initial GI and IAI (*n* = 48, *r* = 0.63, *p* < 0.001). Multivariate regression analysis showed that IAI was a significant independent determinant of GI (*β* = 2.69, *p* = 0.010; Table 2).

**TABLE 2:** Univariate and multiple regression analyses for the initial GI.

Variables	Univariate analysis		Multiple regression analysis	
	Standardized *β*	*p*	Standardized *β*	*p*
Age (years)	−0.35	0.014	−1.06	0.291
Male	0.23	0.104		
Height (cm)	0.38	0.007		
Weight (kg)	0.48	0.001		
BMI (kg/m^2^)	0.40	0.005	0.36	0.722
CHADS_2_ score	−0.03	0.856		
Heart failure	−0.09	0.532		
Hypertension	−0.09	0.540		
Diabetes mellitus	−0.13	0.358		
Stroke	0.09	0.507		
Blood chemistry				
Hb (g/dL)	0.36	0.010	0.74	0.465
BUN (mg/dL)	0.01	0.962		
Cre (mg/dL)	0.02	0.857		
Na (mEq/L)	−0.43	0.002	−0.63	0.532
HbA1c (%)	0.17	0.235		
BNP (pg/mL)	−0.46	0.001	−1.99	0.052
Echocardiography				
LVEF (%)	0.15	0.298		
LAD (mm)	−0.09	0.494		
LA volume (mL)	−0.02	0.881		
PF volume (cm^3^)	0.39	0.006	−0.12	0.904
IAI (Ω)	0.63	< 0.001	2.69	0.010

Abbreviations: BMI, body mass index; BNP, brain natriuretic peptide; BUN, blood urea nitrogen; Cre, creatinine; GI, generator impedance; Hb, hemoglobin; HbA1c, glycated hemoglobin; IAI, intracardiac atrial impedance between the right atrium and the coronary sinus; LA, left atrial; LAD, left atrial dimension; LVEF, left ventricular ejection fraction; Na, sodium; PF, pericardial fat.

### 3.3 Factors Influencing IAI

To identify factors influencing IAI, multivariate regression analysis adjusted for potential confounders was performed (Table 3). The result showed that the PF volume (*β* = 0.45, *p* < 0.001), sodium (*β* = −0.26, *p* = 0.001), and Hb values (*β* = 0.23, *p* = 0.015) were significant predictors of IAI, but not BMI.

**TABLE 3:** Univariate and multiple regression analyses for the IAI.

Variables	Univariate analysis		Multiple regression analysis	
	Standardized *β*	*p*	Standardized *β*	*p*
Age (years)	−0.26	0.008	−0.15	0.072
Male	0.30	0.002	−0.14	0.166
Height (cm)	0.24	0.014		
Weight (kg)	0.41	< 0.001		
BMI (kg/m^2^)	0.42	< 0.001	0.04	0.641
CHADS_2_ score	0.04	0.699		
Heart failure	0.02	0.817		
Hypertension	0.11	0.265		
Diabetes mellitus	0.21	0.029	0.04	0.638
Stroke	−0.04	0.672		
Blood chemistry				
Hb (g/dL)	0.39	< 0.001	0.23	0.015
BUN (mg/dL)	0.16	0.111		
Cre (mg/dL)	0.12	0.220		
Na (mEq/L)	−0.33	0.001	−0.26	0.001
HbA1c (%)	0.16	0.096		
BNP (pg/mL)	−0.15	0.127		
Echocardiography				
LVEF (%)	−0.01	0.885		
LAD (mm)	0.01	0.973		
LA volume (mL)	−0.09	0.323		
PF volume (cm^3^)	0.57	< 0.001	0.45	< 0.001

Abbreviations: BNP, brain natriuretic peptide; BUN, blood urea nitrogen; Cre, creatinine; Hb, hemoglobin; HbA1c, glycated hemoglobin; IAI, intracardiac atrial impedance; LA, left atrium; LAD, left atrial dimension; LVEF, left ventricular ejection fraction; Na, sodium; PF, pericardial fat.

### 3.4 Association Between the PF Volume and IAI

**FIGURE 4:**
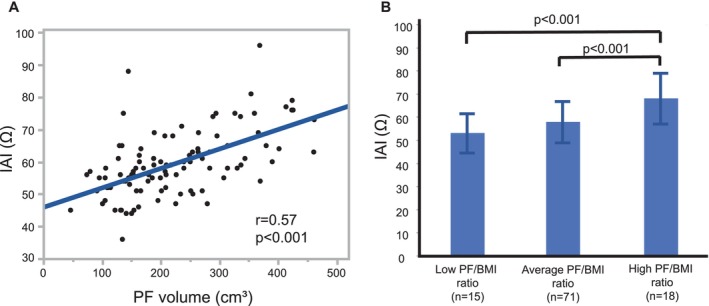
Relationship between the IAI and PF volume. (A) The IAI was positively correlated with the PF volume (*n* = 104, *r* = 0.57, *p* < 0.001). (B) The patients were categorized into three groups based on their PF/BMI ratio. The high PF/BMI group had the highest IAI (68.3 ± 10.9 Ω), which was significantly higher than the low PF/BMI (53.3 ± 8.5 Ω, *p* < 0.001) and average PF/BMI groups (58.2 ± 8.9 Ω, *p* < 0.001). BMI, body mass index; IAI, intracardiac atrial impedance; PF, pericardial fat.

Figure 4A shows a significant positive correlation between the IAI and PF volume (*n* = 104, *r* = 0.57, *p* < 0.001). When stratified by AF type, significant positive correlations between the IAI and PF volume were observed in both the PAF group (*n* = 42, *r* = 0.51, *p* = 0.001) and PeAF group (*n* = 52, *r* = 0.61, *p* < 0.001) (Figure S1). Although IAI did not differ between the PAF and PeAF groups (58.3 ± 9.7 vs. 59.1 ± 9.9 Ω, *p* = 0.706), PF volume was significantly higher in the PeAF group than in the PAF group (235.3 ± 98.4 versus 187.3 ± 78.3 cm^3^, *p* = 0.014) (Table S1).

Furthermore, the PF volume was positively correlated with BMI (*r* = 0.52, *p* < 0.001), indicating the PF volume may be affected by BMI. To clarify this possibility, the patients were categorized into three groups based on their PF/BMI ratio, with a mean ratio of 8.72 ± 3.41. The low PF/BMI ratio group (*n* = 15) comprised individuals with values below one SD from the mean; the average PF/BMI ratio group (*n* = 71) comprised individuals within one SD; and the high PF/BMI ratio group (*n* = 18) comprised individuals with values above one SD. Among these, the high PF/BMI ratio group exhibited the highest IAI (68.3 ± 10.9 Ω), which significantly exceeded both the low PF/BMI ratio group (53.3 ± 8.5 Ω, *p* < 0.001) and the average PF/BMI ratio group (58.2 ± 8.9 Ω, *p* < 0.001; Figure 4B). When stratified by AF type, there were no significant differences in IAI between the PAF and PeAF groups within each PF/BMI category (Figure S2).

### 3.5 Factors Associated With PF/BMI Ratio

The PF/BMI ratio was significantly higher in the PeAF group than in the PAF group (9.1 ± 3.4 vs. 7.6 ± 2.8, *p* = 0.032) (Table S1). To identify factors associated with the PF/BMI ratio, a multivariate regression analysis adjusted for potential confounders was performed in 94 patients with AF. Male sex was identified as a significant independent predictor of PF/BMI ratio (*β* = 0.32, *p* = 0.016) (Table 4).

**TABLE 4:** Univariate and multiple regression analyses for the PF/BMI ratio.

Variables	Univariate analysis		Multiple regression analysis	
	Standardized *β*	*p*	Standardized *β*	*p*
Age (years)	−0.06	0.572		
Male	0.50	< 0.001	0.32	0.016
PeAF	0.22	0.032	0.04	0.614
Height (cm)	0.44	< 0.001		
Weight (kg)	0.40	< 0.001		
BMI (kg/m^2^)	0.26	0.013		
CHADS_2_ score	0.22	0.031	0.10	0.402
Heart failure	0.06	0.536		
Hypertension	0.26	0.011	0.10	0.302
Diabetes mellitus	0.24	0.018	0.12	0.249
Stroke	0.08	0.467		
Blood chemistry				
Hb (g/dL)	0.36	0.001	0.11	0.336
BUN (mg/dL)	0.08	0.443		
Cre (mg/dL)	0.39	0.001	0.07	0.506
Na (mEq/L)	−0.21	0.044	−0.13	0.149
HbA1c (%)	0.14	0.176		
BNP (pg/mL)	−0.01	0.942		
HDL‐C (mg/dL)	−0.21	0.040	−0.11	0.251
TG (mg/dL)	0.18	0.086		
Echocardiography				
LVEF (%)	−0.13	0.199		
LAD (mm)	0.19	0.071		
LA volume (mL)	0.02	0.848		

Abbreviations: BMI, body mass index; BNP, brain natriuretic peptide; BUN, blood urea nitrogen; Cre, creatinine; Hb, hemoglobin; HbA1c, glycated hemoglobin; HDL‐C, high‐density lipoprotein cholesterol; LA, left atrial; LAD, left atrial dimension; LVEF, left ventricular ejection fraction; Na, sodium; PeAF, persistent atrial fibrillation; PF, pericardial fat; TG, triglyceride.

## 4 Discussion

This study demonstrated a significant correlation between the initial GI and IAI, with IAI emerging as an independent determinant of GI by the multivariate regression analysis. Furthermore, we identified PF volume, and serum Na and Hb levels as significant factors influencing IAI, providing valuable insights into the role of anatomical factors and blood composition in shaping impedance dynamics during AF ablation. While this correlation might be physiologically expected, the clinical utility of IAI is rooted in its high reproducibility and stability. Our data and clinical observations suggest that IAI serves as a consistent, patient‐specific metric that remains stable even across different stages of the disease or following repeated procedures. Unlike GI, which is a dynamic and reactive value influenced by real‐time procedural factors (e.g., catheter contact and irrigation), IAI measured via the BeeAT catheter provides a “clean” baseline of the patient's electrical substrate. This allows for the pre‐procedural identification of high‐impedance patients, enabling a more tailored and proactive approach to RF power management and the prevention of complications like steam pops. Thus, IAI provides a novel and practical screening tool that bridges the gap between anatomical assessment and real‐time ablation dynamics.

The positive correlation between IAI and PF volume suggests that the PF, a known contributor to cardiovascular pathology, plays a critical role in influencing tissue impedance. Due to its non‐conductive nature, the PF is likely to increase the resistance in the impedance circuit, resulting in a higher IAI. Previous studies have linked increased PF to adverse cardiac outcomes, including AF recurrence and atrial fibrosis [4, 9]. Our findings build on this body of knowledge by demonstrating that PF not only affects the electrophysiological properties of the atria but also contributes to the mechanical properties that can be quantified using impedance measurements during ablation procedures.

A key finding of this study is the significant correlation between IAI and PF volume in both PAF and PeAF groups. While IAI is measured using intra‐cardiac electrodes, it does not exclusively reflect the impedance of the blood pool. According to volume conductor theory, the electric field generated between the multipolar RA and CS electrode arrays permeates the myocardial wall and encompasses the surrounding epicardial space. Adipose tissue is known to have significantly higher electrical resistivity compared to blood or myocardium, effectively acting as an electrical insulator [10]. Therefore, an increase in PF volume surrounding the heart increases the total impedance within the measurement circuit. The robustness of this relationship is further supported by our multivariable analysis, in which PF remained a significant predictor of IAI while BMI did not. This indicates that IAI specifically captures the localized insulating effect of pericardiac fat rather than reflecting systemic obesity. The use of a multi‐electrode array in the BeeAT catheter likely enhances this sensitivity by creating a broader sensing volume, allowing IAI to serve as a reliable surrogate marker for local epicardial adiposity.

The observed relationships between IAI and serum Na and Hb levels highlight the importance of blood components in the modulation of tissue conductivity. Na ions, which are highly conductive, inversely influence impedance, whereas Hb, a major component of red blood cells, increases impedance due to its effect on tissue composition and fluid conductivity [11]. These findings suggest that electrolyte imbalances and variations in Hb levels may have a direct impact on the success and safety of RF ablation and warrant consideration during the preoperative evaluation.

The relationship between the PF volume and BMI was also of interest. While BMI is often used as a surrogate marker for body fat distribution, our study demonstrated that PF volume has a more direct and significant correlation with IAI than BMI. This distinction is important because patients with a lower BMI but higher PF volume may present with elevated IAI, suggesting that BMI alone is not sufficient to assess the risk factors associated with impedance variability during ablation. The categorization of patients based on their PF/BMI ratio further supports this notion, with the high PF/BMI ratio group showing a significantly higher IAI than the low and average PF/BMI groups, underscoring the importance of PF in determining impedance levels. Furthermore, our study demonstrated that although PF volume and PF/BMI ratio were significantly higher in the PeAF group than in the PAF group (Table S1), no significant difference in IAI was observed between the PAF and PeAF groups within each PF/BMI category (Figure S2). Moreover, multivariable regression analysis revealed that only male sex was a significant independent predictor for PF/BMI ratio, but not PeAF (Table 4). Previous studies have reported sex differences in visceral fat volume and PF volume [12, 13]. In male patients, it may be possible that the IAI, which can be easily measured during ablation, could be used to identify cases in which PF is more prevalent than BMI.

From a clinical perspective, these findings have important implications for optimizing AF ablation procedures. By understanding the factors that influence GI and IAI, clinicians can better predict and adjust for variations in tissue impedance, thereby potentially improving the precision and efficacy of RF. The use of IAI as a predictive marker of GI could serve as a valuable tool for real‐time monitoring of impedance during ablation, allowing dynamic adjustments to energy delivery based on patient‐specific anatomical and physiological characteristics.

### 4.1 Limitations

Despite the promising findings of this study, several limitations should be acknowledged. First, the study was conducted at a single center, which may limit the generalizability of the findings. The relatively small sample size, particularly for the subgroup analysis of GI, may have reduced the statistical power to detect more nuanced associations. The retrospective nature of this study precludes the establishment of causal relationships between the identified factors and impedance measurements. Although the study used advanced imaging techniques to assess PF volume, we did not examine other potentially relevant parameters, such as body fat distribution or visceral fat content, which may have confounded the results. Finally, the study did not evaluate long‐term outcomes of the ablation procedures, such as arrhythmia recurrence, which would provide further insight into the clinical relevance of the observed impedance changes. Future prospective multicenter studies with larger sample sizes are needed to validate these findings and to explore the clinical utility of IAI as a tool for real‐time ablation monitoring and adjustment.

## 5 Conclusions

This study showed that IAI is significantly influenced by PF volume, serum Na level, and Hb concentration. Furthermore, IAI was found to be an independent determinant of GI during AF ablation procedures. These findings suggest that the IAI not only reflects the anatomical and physiological variability in patients but may also serve as a valuable real‐time indicator for predicting GI, allowing clinicians to adjust ablation strategies accordingly. The observed correlations among PF, blood components, and IAI underscore the importance of individualized patient assessment in optimizing ablation outcomes. However, further research is necessary to fully elucidate the role of IAI as a predictive marker and explore its broader clinical implications for improving the efficacy and safety of AF ablation.

## Funding

The authors have nothing to report.

## Ethics Statement

This study was conducted in accordance with the principles outlined in the Declaration of Helsinki and was approved by the Institutional Review Board of Hirosaki University Graduate School of Medicine (2024‐125).

## Consent

Informed consent was obtained via an opt‐out process.

## Conflicts of Interest

Dr. Masaomi Kimura is an associate professor of the Department of Advanced Management of Cardiac Arrhythmias, which is an endowment Department supported by Medtronic Japan Co. Ltd., and Japan Lifeline Co. Ltd. Dr. Shingo Sasaki received a research grant from Boston Scientific Japan Co. Ltd. and is a concurrent associate professor of the Department of Advanced Management of Cardiac Arrhythmias and the Department of Cardiac Remote Management System, which is an endowment Department supported by BIOTRONIK Japan Co. Ltd. Dr. Hirofumi Tomita received a research grant from Abbott Medical Japan LLC., and is a concurrent professor of the Department of Advanced Management of Cardiac Arrhythmias, the Department of Cardiac Remote Management System, and the Department of Advanced Therapeutics for Cardiovascular Diseases, which is an endowment Department supported by Boston Scientific Japan Co. The other authors declare no conflicts of interest.

## Supporting information


**Figure S1:** Relationship between the intracardiac atrial impedance (IAI) and pericardial fat (PF) volume. The IAI was positively correlated with the PF volume in paroxysmal atrial fibrillation (PAF) group (A) and in persistent atrial fibrillation (PeAF) group (B).
**Figure S2:** Comparison of IAI between PAF and PeAF groups stratified by PF/BMI ratio. Patients were categorized into three groups according to their PF/BMI ratio. There were no significant differences in IAI between the PAF and PeAF groups within each PF/BMI category. IAI indicates intracardiac atrial impedance; PF, pericardial fat; BMI, body mass index; PAF, paroxysmal atrial fibrillation; PeAF, persistent atrial fibrillation.
**Table S1:** Comparison of IAI, PF volume, and PF/BMI ratio between the PAF and PeAF groups.

## Data Availability

The data that support the findings of this study are available on request from the corresponding author. The data are not publicly available due to privacy or ethical restrictions.
