# Supplementary material for: Impact of Pericardial Fat on Intracardiac Atrial Impedance and Generator Impedance
Source: J Arrhythm. 2026 May 27;42(3):e70375. doi: 10.1002/joa3.70375 (PMC13240263; doi:10.1002/joa3.70375)
Supplement: Supplementary file 1 — Figure S1: Relationship between the intracardiac atrial impedance (IAI) and pericardial fat (PF) volume. The IAI was positively correlated with the PF volume in paroxysmal atrial fibrillation (PAF) group (A) and in persistent atrial fibrillation (PeAF) group (B). Figure S2: Comparison of IAI between PAF and PeAF groups stratified by PF/BMI ratio. Patients were categorized into three groups according to their PF/BMI ratio. There were no significant differences in IAI between the PAF and PeAF groups within each PF/BMI category. IAI indicates intracardiac atrial impedance; PF, pericardial fat; BMI, body mass index; PAF, paroxysmal atrial fibrillation; PeAF, persistent atrial fibrillation. Table S1: Comparison of IAI, PF volume, and PF/BMI ratio between the PAF and PeAF groups. [file JOA3-42-e70375-s001.docx]

**Supporting Information**

**Impact of Pericardial Fat on Intracardiac Atrial Impedance and Generator Impedance**

Shogo Hamaura, MD, Masaomi Kimura, MD, PhD, Yuichi Toyama, MD, PhD, Kimitaka Nishizaki, MD, PhD, Takahiko Kinjo, MD, PhD, Yuji Ishida, MD, PhD, Taihei Itoh, MD, PhD, Shingo Sasaki, MD, PhD, Hirofumi Tomita, MD, PhD.

**Figure S1.**

**
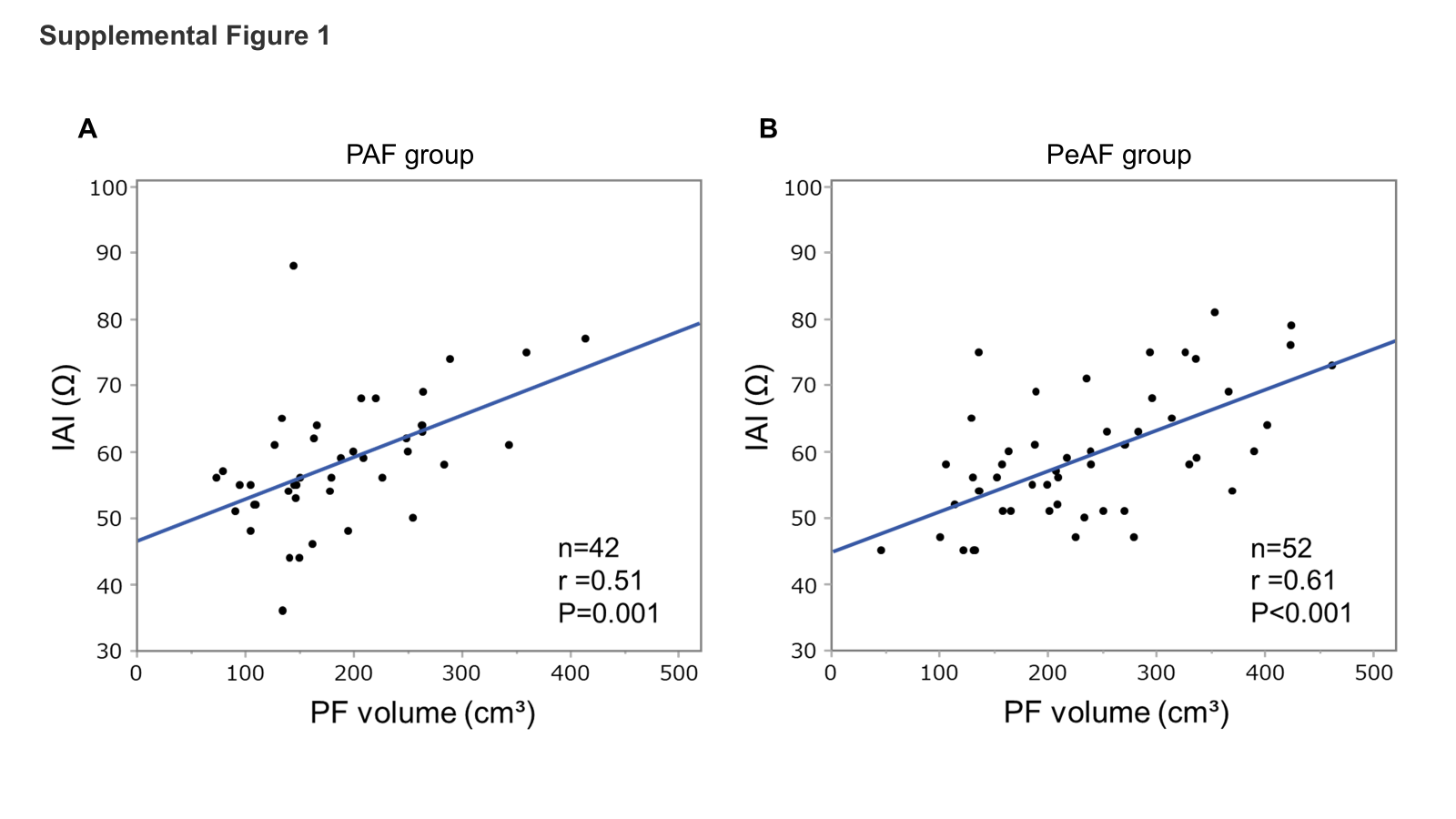
**

**Figure S1.** Relationship between the intracardiac atrial impedance (IAI) and pericardial fat (PF) volume. The IAI was positively correlated with the PF volume in paroxysmal atrial fibrillation (PAF) group (A) and in persistent atrial fibrillation (PeAF) group (B).

**Figure S2.**

**
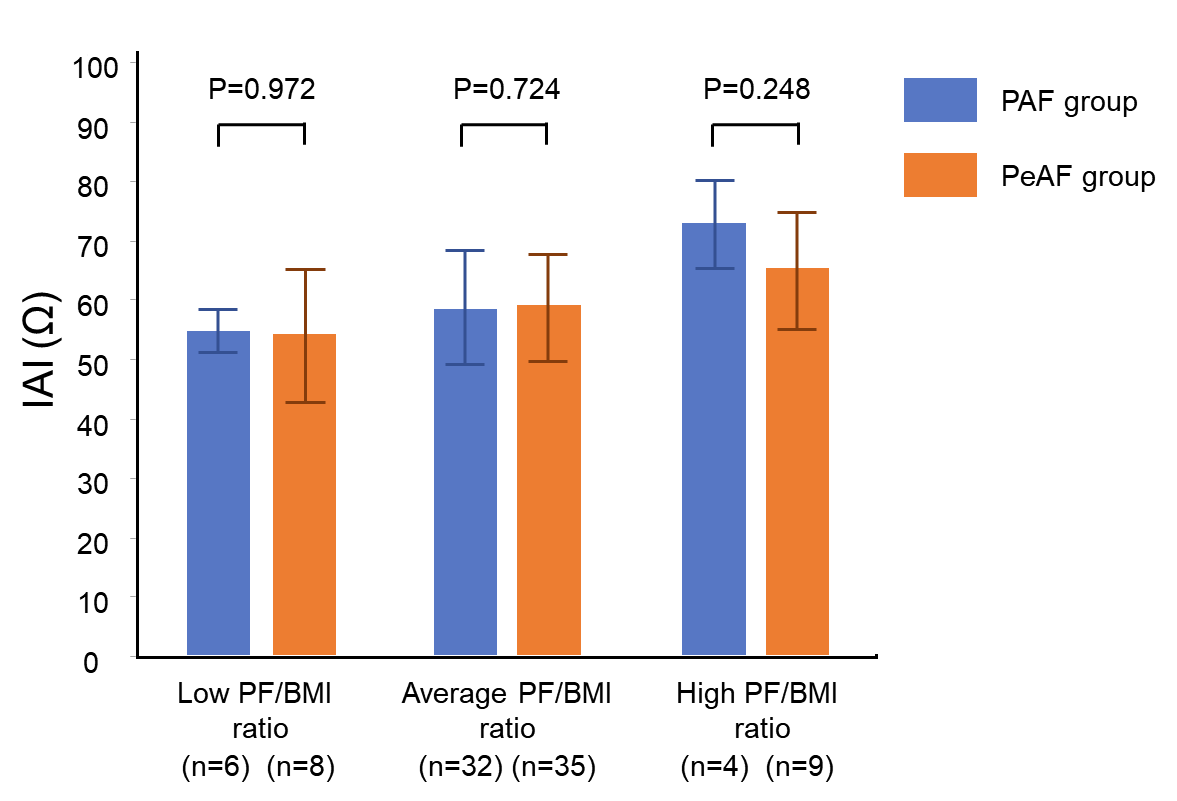
**

**Figure S2.** Comparison of IAI between PAF and PeAF groups stratified by PF/BMI ratio. Patients were categorized into three groups according to their PF/BMI ratio. There were no significant differences in IAI between the PAF and PeAF groups within each PF/BMI category. IAI indicates intracardiac atrial impedance; PF, pericardial fat; BMI, body mass index; PAF, paroxysmal atrial fibrillation; PeAF, persistent atrial fibrillation.

**Table S1.** Comparison of IAI, PF volume, and PF/BMI ratio between the PAF and PeAF groups

| Variables | PAF (n=42) | PeAF (n=52) | P-value |
| --- | --- | --- | --- |
| IAI (Ω) | 58.3±9.7 | 59.1±9.9 | 0.706 |
| PF volume (cm³) | 187.3±78.3 | 235.3±98.4 | 0.014 |
| PF/BMI ratio | 7.6±2.8 | 9.1±3.4 | 0.032 |

BMI indicates body mass index, IAI, intracardiac atrial impedance; PAF, paroxysmal atrial fibrillation; PeAF, persistent atrial fibrillation; PF, pericardial fat.
